# Supplementary material for: Design Optimization and Tradeoff Analysis of an Actuated Continuum Probe for Pulmonary Nodule Localization and Resection
Source: Bioengineering (Basel). 2024 Apr 24;11(5):417. doi: 10.3390/bioengineering11050417 (PMC11118073; doi:10.3390/bioengineering11050417)
Supplement: Supplementary file 1 [file bioengineering-11-00417-s001.zip › bioengineering-2895753-supplementary.pdf]

bioengineering

Supplementary Materials: The following supporting information can be downloaded at: www.mdpi.com/xxx/s1, Figure S1: Design variables vs. objective function cluster plots for the Trial 1 design space.; Figure S2: Three-dimensional design space cluster plots for each design variable vs. objective functions with color bar used to quantify the variable values, where red represents higher values and dark blue represents lower values.

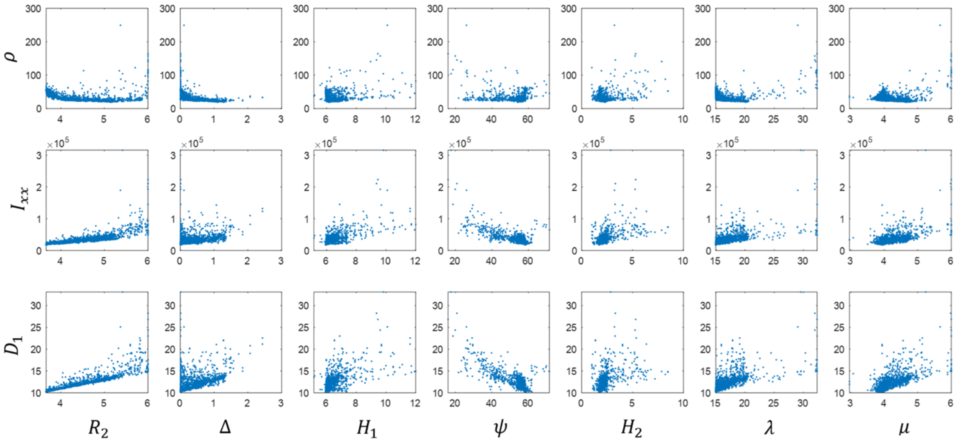

Figure S1. Design variables vs. objective function cluster plots for the Trial 1 design space.

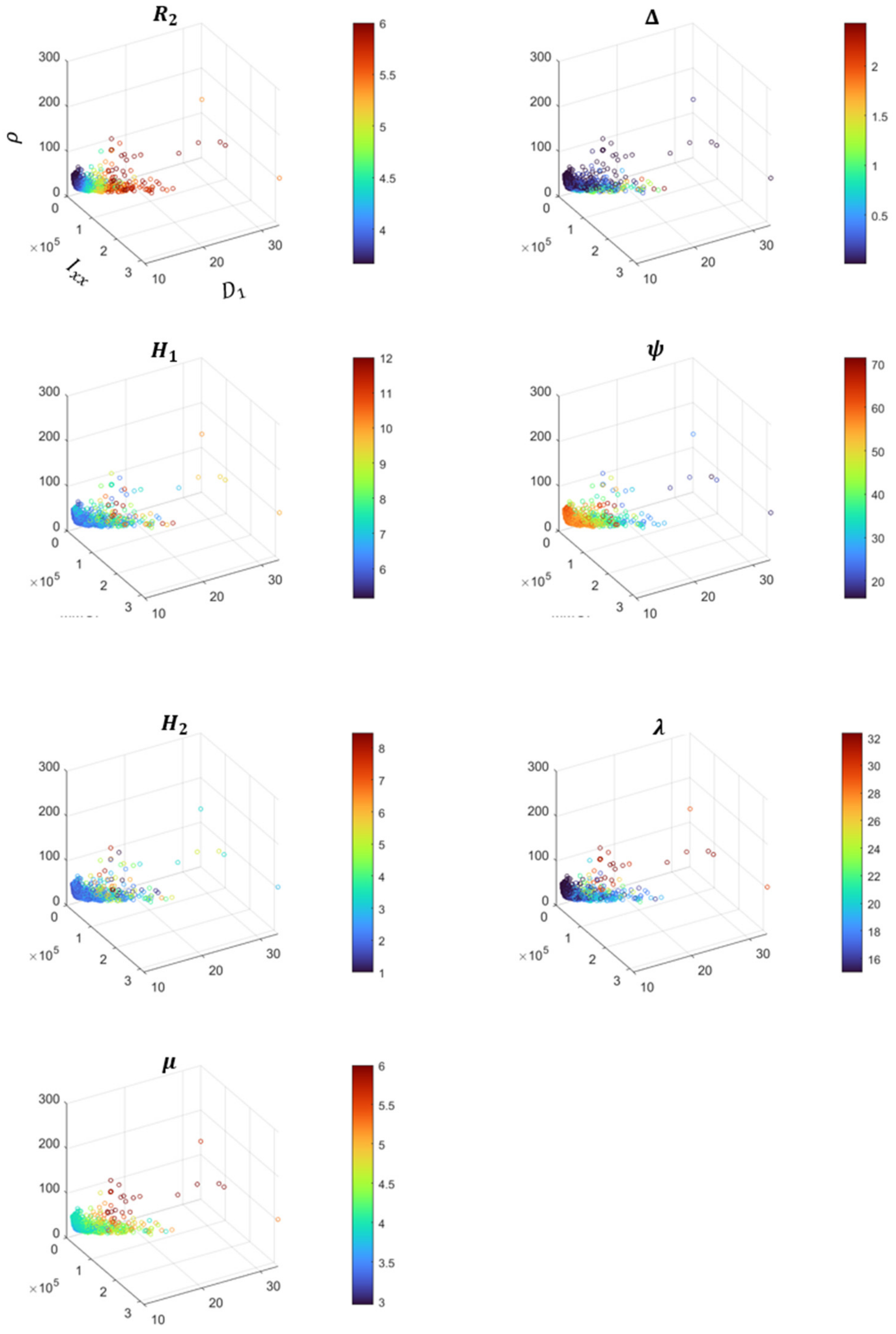

Figure S2. Three-dimensional design space cluster plots for each design variable vs. objective functions with color bar used to quantify the variable values, where red represents higher values and dark blue represents lower values.
